# Failure is a Failure If You Learn Nothing from It

**DOI:** 10.1055/a-2259-0524

**Published:** 2024-02-28

**Authors:** Joon Pio Hong, and Editor-in-Chief Archives of Plastic Surgery

**Affiliations:** 1 Asan Medical Center, University of Ulsan, Ulsan, South Korea

**Figure:**
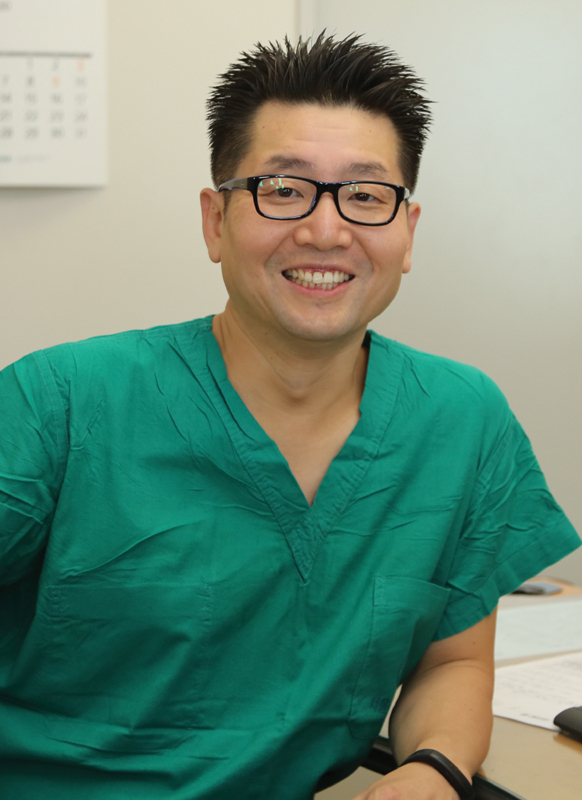
Joon Pio Hong, MD, PhD, MMM, Editor-in-Chief Archives of Plastic Surgery

As surgeons, we perform routine surgeries but also complex surgeries demanding thinking that goes beyond what we have learned. The result of the surgery is an integration of knowledge, technical skills, experience, team dynamics, communication, patient condition, anesthesia, surgical equipment, and many other factors. This is why surgery is often referred to as an “art.” It is like creating, combining, erasing, and choosing colors from the palette. Like any artist, surgeons can create a masterpiece, mediocracy, and failures. Although the risk of failure may exist the same as art, the surgical failure can mean life and death to the patient. This is why it can be harder to cope with failures for surgeons. All surgeons fail at some point in their career. It is how one copes with failures that define a surgeon who will be creating that future masterpiece.

A wise man once said, failure leads to growth. Recognizing and accepting failure takes courage. And this is especially true in addressing failure to patients. Although it is a clinical outcome for the surgeon, it is a deeply personal experience to the patient. The surgeon must always have empathy and be honest to the patient. 1 The surgeons need to be clear in the patient's expectations and warn the possible complications involved and finally be straightforward in managing and communicating the failure. It is never an easy task to admit and converse as we were taught not to fail and even have seen cases of stigmatism. But if one wants to learn from failures, the first step is admitting to oneself of failure. Only then, one can start to think what went wrong or what factors were missed. This is how we can use failures to grow.

Opening to a small forum to discuss and identify the causes that may have led to failures is a model example. Understanding and providing a solution to the problem may be the path to new insights. 2 3 4 One needs to be comfortable to share the problem, embrace constructive criticism, and most of all learn from the failure. This experience will make one humble, realize that there is always a better way, and help stay in the path to solve and perform difficult cases. The real failure is when one becomes discouraged, loses interest, avoids these difficult cases, and most of all ends in giving up the field. I have seen many surgeons walk this path where instead of taking up the challenge, take a detour and leave the field entirely.

Surgical failure is tough for any surgeon. But ironically to avoid future failures, we must continue to make mistakes and learn from it. This is how the field of medicine evolves. Build an environment where one can share failures and mistakes. This will not only help the individual grow, but you will witness the growth as a department or a unit. Failure will come unintentionally but to choose to grow from failures can be made only intentionally. This is the pathway to become a maestro.
